# Prevalence of pelvic floor disorders in adult women being seen in a primary care setting and associated risk factors

**DOI:** 10.1038/s41598-022-13501-w

**Published:** 2022-06-14

**Authors:** Kimberly A. Kenne, Linder Wendt, J. Brooks Jackson

**Affiliations:** Institute for Clinical and Translational Science, University of Iowa Hospitals and Clinics, 200 Hawkins Drive, 31674 PFP, Iowa City, IA 42240 USA

**Keywords:** Urogenital diseases, Quality of life, Weight management

## Abstract

Determine the prevalence of pelvic floor disorders (PFD) stratified by age, race, body mass index (BMI), and parity in adult women attending family medicine and general internal medicine clinics at an academic health system. The medical records of 25,425 adult women attending primary care clinics were queried using International Classification of Diseases-10th Revision codes (ICD-10 codes) for PFD [urinary incontinence (UI), pelvic organ prolapse (POP), and bowel dysfunction (anal incontinence (AI) and difficult defecation)]. Prevalence and odds ratios were calculated using univariate and multivariate analysis for age, race, BMI, and parity when available. Multivariate logistic regression models were used to assess the impact of age, race, BMI, and parity on the likelihood of being diagnosed with a PFD. A separate model was constructed for each of the three PFD categories (UI, POP, and bowel dysfunction) as well as a model assessing the likelihood of occurrence for any type of PFD. The percentage of women with at least one PFD was 32.0% with bowel dysfunction the most common (24.6%), followed by UI (11.1%) and POP (4.4%). 5.5% had exactly two PFD and 1.1% had all 3 categories of PFD. Older age and higher BMI were strongly and significantly associated with each of the three PFD categories, except for BMI and prolapse. Relative to White patients, Asian patients were at significantly lower risk for each category of PFD, while Black patients were at significantly lower risk for UI and POP, but at significantly greater risk for bowel dysfunction and the presence of any PFD. Higher parity was also significantly associated with pelvic organ prolapse. Using multivariate analyses, age, race, and BMI were all independently associated with PFD. PFD are highly prevalent in the primary care setting and should be screened for, especially in older and obese women. BMI may represent a modifiable risk factor.

## Introduction

Pelvic floor disorders (PFD) which include urinary incontinence (UI), pelvic organ prolapse (POP) and bowel dysfunction (difficult defecation and anal incontinence (AI)) are common among adult women. Nygaard et al. found that approximately one quarter of women in the United States have at least 1 PFD, with that rate more than doubling for women older than 80 years^1^. These disorders impact quality of life for many women. A true prevalence of these disorders is difficult to capture. Differences in definition, both in clinical practice and in the literature create variability in estimates of prevalence and incidence^2^. The population being evaluated (treatment seeking population versus general population) as well as how the presence of these disorders is evaluated (validated questionnaire, physical exam, or review of the medical record) impacts the reported prevalence of these disorders.

Regardless of how prevalence is determined, studies have consistently demonstrated risk factors for PFD include increasing age (menopausal status), prior hysterectomy, vaginal birth, obesity, smoking, and inheritance of a connective tissue disorder^3^. More specifically, Blomquist et al. demonstrated the cumulative incidence of each pelvic floor disorder was significantly associated with mode of delivery^4^. Compared with spontaneous vaginal delivery, cesarean delivery was significantly associated with a lower hazard of stress urinary incontinence (SUI), overactive bladder (OAB), and POP while an operative vaginal delivery was significantly associated with a higher hazard of AI and POP^4^.

The US population is projected to become increasingly elderly, overweight, and obese. The proportion of the population aged 65 years and older is expected to double between 2010 and 2050^5^. By 2030, nearly 1 in 2 adults will be obese (48.9%; 95% confidence interval [CI], 47.7 to 50.1), and nearly 1 in 4 adults is projected to have severe obesity (24.2%; 95% CI 22.9–25.5)^6^. These projected changes will likely further increase the burden and cost of PFD on society and the healthcare system.

In order to examine the prevalence of PFD and associations with age, race, body mass index (BMI), and parity in a population of adult women attending family medicine and general internal medicine clinics at an academic health system, we obtained patient demographic and clinical data from the electronic medical record for over 20,000 adult female patients who were living and seen in the last 5 years. The primary objective was to determine prevalence of PFD in a population being seen in a primary care setting. We chose to query family medicine and general internal medicine clinics as prevalence data tend to reflect a treatment seeking population or the general population at large and are lacking from a primary care setting specifically. Data reflective of this population may help primary care providers improve screening and referral for PFD which are important quality of life concerns for many adult women.

## Methods

Living adult (≥ 18 years old) female patients seen at the University of Iowa Hospitals and Clinics in the family medicine or general internal medicine clinic in the last 5 years were included. Patient age, race, BMI, parity (when available), and disease conditions were obtained from the Epic Systems electronic health record. Health records were queried for the presence of PFD using ICD-10 codes (stress urinary incontinence: N39.3, urge urinary incontinence: N39.41, other incontinence: R32, N39.46, N39.43, N39.44, N39.45, N39.490, R39.81, pelvic organ prolapse: N81.9, N81.82, N81.83, N81.84, N81.85, N81.89, N81.11, N81.12, N81.10, N81.6, N81.81, N81.2, N81.3, N81.4, N99.3, N81.5, difficult defecation: K59.00, K59.01, K59.02, K59.09, and anal incontinence: R15.9). Obstetrics & gynecology and urology clinics were excluded from data collection to minimize sampling bias.

Deidentified patient information was analyzed from most recent patient visit using multivariate logistic regression to assess the relationship between PFD and each risk factor; age, race, BMI, and parity in a subset of women for which these data were available. For each PFD category, including presence of any PFD, odds ratio estimates for risk factors (age, race, BMI and parity when stated) were obtained from multivariate models that include each of these variables. Confidence intervals and p-values were also calculated from these models.

Categories of BMI were as follows: healthy weight 18.5–24.99, overweight 25.0–29.99, class 1 obesity 30.0–34.99, class 2 obesity 35.0–39.99, class 3 obesity ≥ 40.0. Patients with a BMI > 90 or < 18.5 were excluded from analysis. Comparisons with p-values < 0.05 were considered significant. All analyses were performed using R, version 4.1.1.

The University of Iowa Institutional Review Board (IRB) review was obtained for this study. All methods were carried out in accordance with the IRB’s guidelines and regulations. Given the retrospective nature of this work the need for informed consent was waived by the IRB.

## Results

25,425 women age ≥ 18 years old, with a BMI between 18.5 and 90, were included. The cohort was predominantly white (19,873 (78.2%)), overweight (mean (SD) BMI 29.42 (8.01)), and middle aged (mean (SD) age 48.44 (18.60)) (Table 1). Overall, 32.0% (8145) of the cohort carry the diagnosis of any PFD. 11.1% (2814) experience some form of UI, 4.0% (1014) experience POP, and 25.0% (2646) experience bowel dysfunction. The most common type of UI was other urinary incontinence (1104 (7.2%)). The most common type of bowel dysfunction was difficult defecation (4014 (15.8%)). In this patient sample, 6.5% (1661) had two or more PFDs (Table 2).

**Table 1:** Cohort demographics.

Characteristic	Full cohort	Cohort with parity data
	N = 25,425^1^	N = 3214^1^
Age	47 (32, 64)	37 (32, 41)
**Race**		
White	19,873 (78)	2007 (62)
Asian	1515 (6.0)	299 (9.3)
Black or African American	2019 (7.9)	537 (17)
Other	2018 (7.9)	371 (12)
**BMI**		
Healthy weight	8807 (35)	1067 (33)
Overweight	6967 (27)	942 (29)
Obese Class 1	4448 (17)	575 (18)
Obese Class 2	2619 (10)	329 (10)
Obese Class 3	2584 (10)	301 (9.4)
**Parity**		
0		1019 (32)
1		1079 (34)
2		617 (19)
3		286 (8.9)
> 3		213 (6.6)

^1^Median (IQR); n (%).

**Table 2:** Prevalence of pelvic floor disorders.

Characteristic	Full cohort	Cohort with parity data
	N = 25,425^1^	N = 3214^1^
Any disorder	8145 (32)	1177 (37)
Multiple disorders	1661 (6.5)	172 (5.4)
Urinary incontinence	2814 (11)	261 (8.1)
Urinary stress	1104 (4.3)	158 (4.9)
Urinary urge	895 (3.5)	30 (0.9)
Other urinary	1835 (7.2)	133 (4.1)
Prolapse	1014 (4.0)	140 (4.4)
Bowels	6246 (25)	975 (30)
Anal incontinence	3029 (12)	528 (16)
Difficulty defecation	4014 (16)	587 (18)

^1^n (%).

In multivariable models, increasing age was associated with any type of PFD (OR 1.021, per one year age, 95% CI 1.020–1.023, p < 0.001). Age also increased the risk for diagnosis of each individual type of PFD; UI (OR 1.045, 95% CI 1.042–1.047, p < 0.001), POP (OR 1.035, 95% CI 1.032–1.039, p < 0.001), and bowel dysfunction (OR 1.010, 95% CI 1.009–1.012, p < 0.001) (Table 3). Of the patients with a diagnosis of a PFD, 29.6% were over age 65 while only 12.2% were under age 30. For each 10-year increase in age, women were 1.236 times more likely to have any PFD.

**Table 3:** Multivariable analysis of clinical characteristics and presence of pelvic floor disorders.

	Any PFD			Urinary Incontinence			Prolapse			Bowel Dysfunction		
	OR^1^	95% CI^1^	p-value	OR^1^	95% CI^1^	p-value	OR^1^	95% CI^1^	p-value	OR^1^	95% CI^1^	p-value
Age	1.02	1.02, 1.02	** < 0.001**	1.04	1.04, 1.05	** < 0.001**	1.03	1.03, 1.04	** < 0.001**	1.01	1.01, 1.01	** < 0.001**
**Race**												
White	—	—		—	—		—	—		—	—	
Asian	0.69	0.60, 0.78	** < 0.001**	0.66	0.51, 0.83	** < 0.001**	0.55	0.36, 0.79	**0.002**	0.68	0.59, 0.79	** < 0.001**
Black or African American	1.36	1.23, 1.50	** < 0.001**	0.62	0.51, 0.75	** < 0.001**	0.48	0.33, 0.68	** < 0.001**	1.63	1.47, 1.80	** < 0.001**
Other	1.08	0.97, 1.19	0.156	0.91	0.76, 1.08	0.293	0.83	0.61, 1.09	0.189	1.20	1.08, 1.33	** < 0.001**
**BMI**												
Healthy Weight	—	—		—	—		—	—		—	—	
Overweight	1.19	1.11, 1.27	** < 0.001**	1.31	1.17, 1.46	** < 0.001**	1.32	1.13, 1.56	** < 0.001**	1.14	1.05, 1.22	** < 0.001**
Obese Class 1	1.20	1.11, 1.30	** < 0.001**	1.61	1.43, 1.83	** < 0.001**	1.23	1.02, 1.49	**0.026**	1.14	1.05, 1.24	**0.002**
Obese Class 2	1.38	1.26, 1.52	** < 0.001**	1.99	1.73, 2.29	** < 0.001**	1.01	0.80, 1.28	0.913	1.20	1.09, 1.33	** < 0.001**
Obese Class 3	1.51	1.37, 1.66	** < 0.001**	2.54	2.21, 2.91	** < 0.001**	0.92	0.71, 1.18	0.509	1.31	1.18, 1.45	** < 0.001**

Significant values are in bold.

^1^OR = Odds Ratio, CI = Confidence Interval.

PFD prevalence varied by reported race. Relative to White patients, Asian patients had a significantly lower risk for any PFD (OR 0.690, 95% CI 0.605–0.785, p < 0.001). Relative to White patients, Black patients were at significantly lower risk for UI (OR 0.618, 95% CI 0.507–0.747, p < 0.001) and POP (OR 0.490, 95% CI 0.335–0.90, p < 0.001), but at significantly greater risk for bowel dysfunction (OR 1.622, 95% CI 1.466–1.793, p < 0.001) and any type of PFD (OR 1.359, 95% CI 1.231–1.499, p < 0.001). This finding is reflective of the high prevalence of bowel dysfunction in this subset. Relative to White patients, patients who did not identify as White, Black, or Asian did not differ significantly in their prevalence of PFDs, except for having a greater rate of bowel dysfunction (OR 1.199, 95% CI 1.077–1.333, p < 0.001) (Table 3).

**Figure 1:**
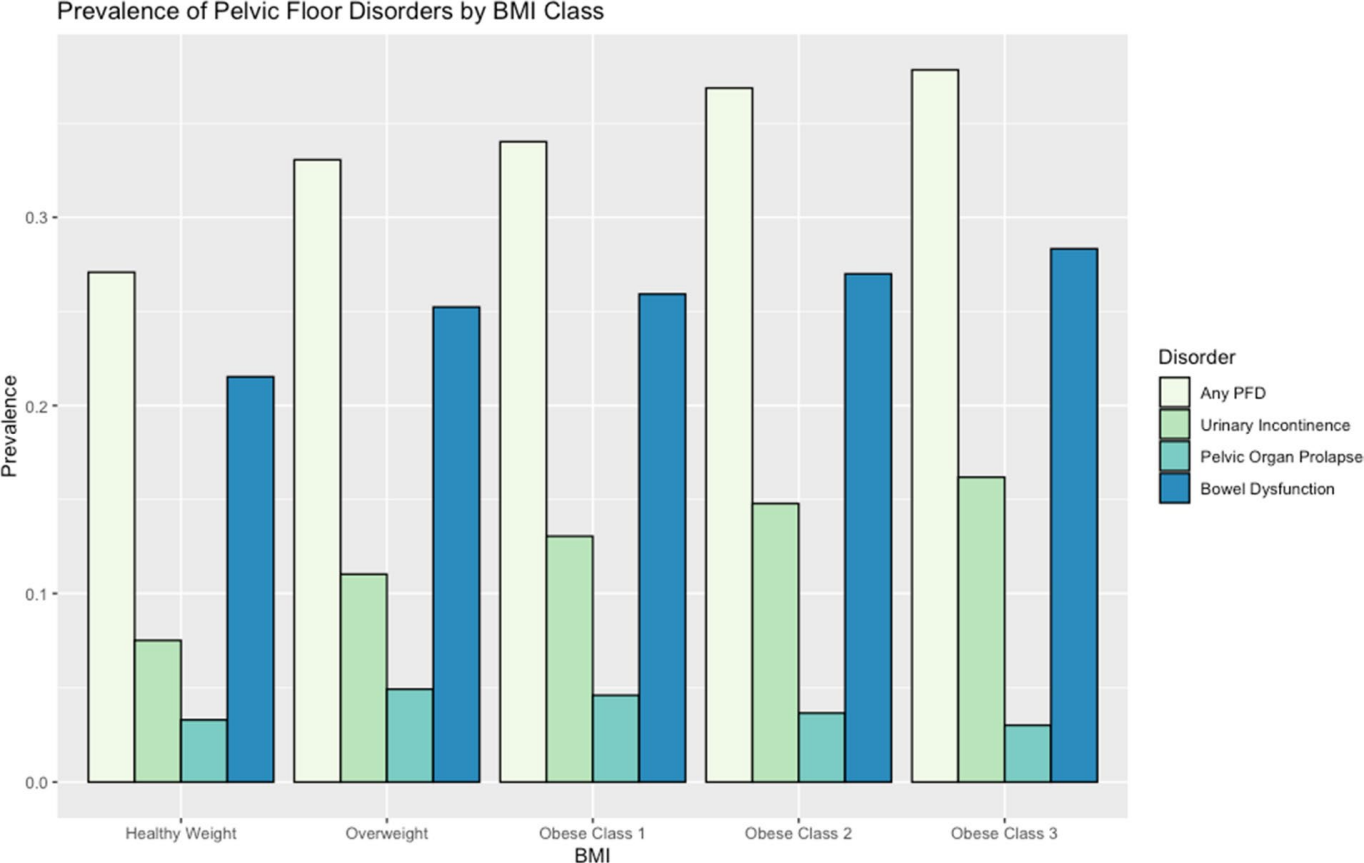
Prevalence of pelvic floor disorders by BMI class.

Increasing BMI was associated with risk of UI (OR 1.038, by one BMI unit, 95% CI 1.033–1.043, p < 0.001), bowel dysfunction (OR 1.012, 95% CI 1.008–1.015, p < 0.001), and any type of PFD (OR 1.018, 95% CI 1.014–1.02, p < 0.001). No relationship was observed between BMI and POP (OR 0.998, 95% CI 0.99–1.006, p = 0.603) (Table 3 and Fig. 1).

Patients with available parity data (3214 (12.6%)) tended to be primiparous (median parity [IQR] 1 [0–2]) and 36.6% of patients in this subset carry the diagnosis of a PFD. Increasing parity was only associated with POP (OR 1.155, 95% CI 1.020–1.300, p = 0.020). 8.1% experience some form of UI, 4.4% experience POP, and 30.3% experience bowel dysfunction. The most common type of UI was stress urinary incontinence (4.9%). The most common type of bowel dysfunction was difficulty with defecation (18.3%). Of patients diagnosed with a pelvic floor disorder for which parity data was available 14.6% carried the diagnosis of two or more PFD.

In this subset, when controlling for age, race, and BMI, parity was not found to increase the odds of diagnosis of general PFD (OR 1.035, 95% CI 0.98–1.098, p = 0.243) or any category of PFD except POP; UI (OR 1.080, 95% CI 0.98–1.187, p = 0.116), POP (OR 1.157, 95% CI 1.022–1.303, p = 0.018), bowel dysfunction (OR 1.035, 95% CI 0.97–1.100, p = 0.269).

## Discussion

In this cohort of women seen in a primary care setting PFD were common with a prevalence of 32.0% based on ICD-10 codes. This finding is similar to previously published work which have found PFD are common^2,7,8^. Of those individuals who experience one PFD, 6.5% experience greater than one. This finding differs from a study of more than 5000 parous Swedish women in which 46% had at least one disorder and almost a third of these symptomatic women had two or more disorders^9^. This is likely due to both the population examined in the Swedish study (all parous women) and the manner in which presence of PFD was determined (PFD specific post-natal questionnaire). In another study, at least one PFD was reported by 34% of women over 40 years of age, and 16% of symptomatic women had more than one disorder; specifically, 9% of the symptomatic women had both UI and AI and 7% had both UI and POP^10^.

The prevalence of PFD increased with age and BMI (except in the case of POP) and were less common in individuals who identify as Asian. Similar to previously published work, PFD become increasingly common as women age. Nygaard et al. found that nearly one-quarter of all women and more than one-third of older women reported symptoms of at least 1 PFD^1^. Hallock et al. recognized both the prevalence and severity of PFD is associated with age and obesity^11^. Wu et al., in a study examining the prevalence and trends of PFD in the US found age, higher BMI, greater parity, and hysterectomy were associated with higher odds of one or more pelvic floor disorder^7^. We also found age increased the risk for any PFD (OR 1.021, 95% CI 1.020–1.023, p < 0.001) as did BMI (OR 1.018, 95% CI 1.014–1.02, p < 0.001).

The potential association of PFD with race is complex with previously published prevalence studies demonstrating mixed results. At present there is no known pathophysiologic reason for differences in the prevalence of PFD based on a woman’s race. We found Asian women had a lower prevalence of PFD. In our cohort, black women had a lower prevalence of UI and POP but a greater prevalence of bowel dysfunction. It is unclear if these differences are reflective of true differences in prevalence or the result of study limitations which are further discussed below. As reported in prior work, prevalence studies which rely on patient reported outcomes or self-reported degree of bother may be biased by culture-based differences in recognition of disease versus normal state^11^. Our results echo that racial and ethnic variations in the prevalence and degree of bother associated with PFD warrant future investigation^1^. We are unable to make specific recommendations on the evaluation of PFD in a primary care population in reference to a woman’s race aside from advising all women be screened for PFD.

Different from previously published work, we did not find an association between POP and BMI (OR 0.998, 95% CI 0.99–1.006, p = 0.603)^12^. We were also unable to demonstrate a relationship with PFD and parity (OR 1.035, 95% CI 0.98–1.097, p = 0.253) except in the case of POP (OR 1.155, 95% CI 1.020–1.300, p = 0.020)^12^. We included both anal incontinence and difficult defecation ICD-10 codes as representative of patients with bowel dysfunction and thus a PFD. Previously published work has mostly examined fecal or anal incontinence alone. This difference may account for bowel dysfunction being the most common PFD in our cohort. We were limited by parity data being available for a small subset of our population.

Strengths of this study include the large sample size and examination of a primary care seeking population rather than a population seeking care in an obstetrics & gynecology or urology clinic. Limitations of this study include dependence on accurate ICD-10 diagnoses coding, accurate recording of other variables (race, BMI), inability to identify parity data for the entire cohort, and inability to confirm diagnoses with direct patient evaluation (validated questionnaires) or examination. Additionally, women seeking care at an academic medical center may not be representative of the general population. Individuals often underreport PFD due to embarrassment, lack of knowledge regarding appropriate provider and treatment options, belief these disorders are physiologic, or prioritizing of other medication conditions in an often short clinic visit in the primary care setting. Reliance on accurate electronic medical records introduces reporting bias both on the part of the patient and the provider. Given the aforementioned barriers for both the patient and primary care provider the actual prevalence of PFD in a primary care setting may actually be higher. We are also unable to ascertain causality or chronicity between risk factors and diagnosis of PFD.

Given the consistent association between age, BMI, and PFD it seems probable the burden of these disorders will continue to increase as the US population becomes increasingly elderly and obese. The age-adjusted prevalence of obesity was 42.4% among adults aged 20 and over in the United States in 2017–2018. The prevalence of severe obesity was higher among women^13^. By 2030 nearly 1 in 2 adults will have obesity (48.9%; 95% confidence interval [CI], 47.7 to 50.1) and nearly 1 in 4 adults is projected to have severe obesity (24.2%; 95% CI 22.9 to 25.5). Nationally, severe obesity is likely to become the most common BMI category among women by 2030^6^. While some factors associated with PFD; age, race, parity, and mode of delivery may represent less modifiable factors, management of obesity may represent an important component of prevention and treatment of PFD. This sentiment is shared among published epidemiologic studies and warrants continued attention^7,11^.

## Conclusion

PFD are prevalent in the primary care setting. Patients should be screened for PFD, especially older and obese women. BMI may be a modifiable risk factor for UI and bowel dysfunction.

## Data Availability

We do not wish to share our dataset however the dataset used and analyzed during the current study is available from the corresponding author on reasonable request with some modification as we do not have consent to share protected health information.
